# Healing of Ligaments and Tendons: Tissue Engineering and Models

**DOI:** 10.3390/ijms232415503

**Published:** 2022-12-07

**Authors:** Gundula Gesine Schulze-Tanzil

**Affiliations:** Institute of Anatomy and Cell Biology, Paracelsus Medical University, Nuremberg and Salzburg, Prof. Ernst Nathan Str. 1, 90419 Nuremberg, Germany; gundula.schulze@pmu.ac.at

The aim of this Special Issue is to summarize the latest developments in tendon/ligament research and tissue engineering (TE), providing helpful approaches for future tendon/ligament reconstruction (Figure 1). 

TE of extracellular matrix (ECM) and collagen-rich tissues such as tendon and ligaments usually requires cell carriers that are biomaterials. Hence, this Special Issue also presents the effects of selected biomaterials on tenogenesis and ligamentogenesis in view of tendon/ligament reconstruction, including decellularized tendons and collagen-functionalized or isotropic nanofibrous polymer scaffolds. For a better understanding of the tendon healing process, novel models are required.

The valuable three-dimensional (3D) in vitro models presented in this Special Issue include biofabricated artificial pathological tendons and versatile mechano-stimulatory approaches to support in vitro teno-/ligamentogenesis [1,2,3]. The effect of the unique microenvironment on tendons/ligaments and their cells requires increasing attention and was, therefore, a major topic of this Special Issue. Accordingly, the effects of an ECM used for tendon TE were influenced by its origin: either harvested from diseased or healthy tendons [3].

Another study investigated the influence of the microenvironment on the tenocyte phenotype using an in vitro indirect co-culture approach (transwell system). Here, the secretome from in vitro statically loaded (Flex cell system) myoblasts induced tenocyte migration, enhanced transition to a myofibroblastic phenotype, and mediated an increase in collagen type I/III ratio [4], suggesting a profound influence on parameters related to the tendon healing process. This shows once again that there is an intimate cross-talk between resident and neighbored cell populations in tendons and surrounding tissue (e.g., muscle and fatty tissue), which also includes important myoblasts in the myotendinous junction of tendons. This cross-talk might also influence intrinsic stem cell populations in the tendon. The cell surface marker CD146 delineates an interfascicular matrix (IFM) resident cell subpopulation in a tendon that is recruited during injury through its ligand laminin-4 [5]. The IFM connects the fascicles of a tendon (Figure 1) and harbors this subpopulation of morphologically distinct cells, which represent pericyte-like progenitor cells, immunoreactive for CD146, which bind laminin-α4 as a specific ligand. Laminin is a natural component of the vascular wall localized at the myotendinous junction and associated with myoblasts [6]. A preclinical model of a rat Achilles tendon shows that these cells were mobilized by traumatic tendon injury accompanied by an increase in laminin-α4. Stem cells are well known to contribute to healing [7]; immature and juvenile tendons might contain more resident stem cells than adult or even aging tendons [8]. In immature tendons, a scarring-free regeneration can be observed [9], but this difference in comparison to adult tendons remains unexplained. Regarding this, the study of Ribitsch et al. [10] presents novel information. The authors studied molecular mechanisms of fetal tendon regeneration in comparison to adult fibrous repair in a sophisticated ovine in utero tendon injury in vivo model [10]. The major difference between the healing in adult and fetal ovine tendons was that, in the tendon defects of adult individuals, a specific and pronounced local immune response characterized by inflammation was detected. This was associated with activated neutrophils, up-regulated pro-inflammatory mediators, neutrophil-attracting chemokines, as well as tissue-damaging antimicrobials, ECM-degrading enzymes, and an oxidative stress response. In contrast, factors secreted by injured fetal tendons included proteins inducing the resolution of inflammation or promoting functional ECM synthesis. Nevertheless, questions remain: Why does this difference exist, and does it depend on the immaturity of the fetal immune system? This study could have great implications for therapy with the idea that resolving factors released by fetal tendons could be therapeutically provided, e.g., by extracellular vesicles (EVs).

EVs represent an emerging field with great hopes and expectations for future therapeutical approaches, but their high variability (size, source, contents) influenced by environmental conditions of cells requires deeper knowledge. Their effect on TE and tendon healing is barely described. Adipose-tissue-derived stem cells (ADSCs) are the most abundant stem cell resource. Chen et al. conducted a detailed analysis on the effects of EVs released by ADSCs and their impact on tendon healing. They found that EVs of ADSCs promoted the healing of traumatized rabbit Achilles tendons [11]. ADSC-derived EVs significantly enhanced the proliferation and migration of tenocytes in vitro. Using the biomechanical properties of a rabbit Achilles tendon defect model, histoarchitecture, and protein synthesis in the injured tendons significantly improved four weeks after treatment with ADSC-derived EVs, with decorin and biglycan being upregulated [11].

Another study included in this Special Issue analyzed the therapeutical effects of different-sized platelet-derived EVs in an in vitro bioengineered tendon disease model [2]. The model combining human-tendon-derived cells with nanofibrous isotropic scaffolds showed the anti-inflammatory, tenogenic and natural remodeling effects of platelet-derived EVs depending on their size. This is a particular advantage, supporting the 3R principle (reduce/replace/refine animal experiments) and translational aspects by working with human cell resources. Another interesting model was presented by combining the cell-free equine tendon ECM of healthy and diseased equine tendons with equine ADSCs, simulating a therapeutical approach that is already clinically practiced in horses. The results show that there was a significantly lower gene expression of enzymes associated with ECM remodeling in the ADSCs colonizing diseased ECM [3]. This should certainly be considered since it could impact the final outcome of the tendon healing process.

Novel models, such as those described above, are required in tendon research. The application of ECM-free approaches or carrier-free ligament 3D models has the advantage of excluding the effects of carriers on cell sources needed for tendon healing or reconstruction. A minispheroid anterior cruciate ligament (ACL) model was presented as a tool for ligament TE, explaining the potential influence factors on cell-free approaches, such as spheroid sizes or assembly techniques [12]. Typical ACL ECM components could be localized in mini-spheroids of all tested sizes confirming their conserved expression profile and their suitability for ligament TE. This spheroid-based approach can be used for scaffold seeding [1]. Cyclic uniaxial stretch significantly increased the scaffold area colonized with ACL fibroblasts as well as tenascin C, connexin 43 and Mohawk gene expression, but also impaired sGAGs and decorin gene expression [1]. A novel healing model that could be used to study the above-mentioned issues was developed by Chen et al. These authors described a tendon-specific double-reporter transgenic mouse that allows the tracking of cell lineages and assessment of functional alterations in vitro and in vivo [13]. This approach could fulfill an unmet requirement of animal models, allowing the real-time monitoring of cell behaviors during tendon development, growth, and repair in vitro and in vivo. It is suitable for analyzing changes in ECM production and cell redifferentiation.

This collection of articles reflects diverse novel approaches undertaken to facilitate tendon/ligament TE and to better understand successful and unsuccessful healing. Nevertheless, many questions remain unanswered. One major question is how to adapt as much of the specific microenvironments as possible (mechanical stimuli, presence of maturation- and disease-associated mediators, immune responses) in novel in vitro tendon/ligament models and how to select ideal resources for EVs, tailoring their contents to optimize tendon healing.

## Figures and Tables

**Figure 1:**
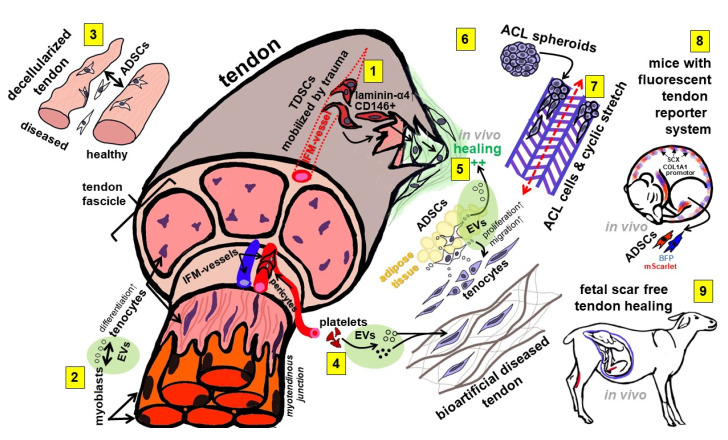
A synopsis of topics investigated in the articles summarized in this Special Issue. 1: CD146+ cells represent an interfascicular stem cell population recruited by laminin-α4 in tendon injury. 2: Mechanostimulated myoblast-derived EVs induce either tenocyte differentiation or myofibroblast transition. 3: ADSCs respond to the decellularized ECM of diseased and healthy tendons with a different remodeling capacity. 4: Platelet-derived EVs exert therapeutic effects in a bioartificial model of a diseased tendon. 5: ADSC-derived EVs mediate tenocyte proliferation as well as migration and stimulate in vivo tendon healing. 6: A mini-spheroid model of ACL ligamentocytes can be used for ACL tissue engineering. 7: Cyclic stretch stabilizes ACL ligamentocyte phenotype in spheroid seeded embroidered scaffolds. 8: A mouse model with tendon-specific reporter genes might allow for the real-time monitoring of tendon healing. 9: A large animal model comparing adult tendon repair with scar-free fetal tendon regeneration points to the differences in the immune response in fetuses and adults. ACL: Anterior cruciate ligament, ADSCs: Adipose-tissue-derived stem cells, COL1A1: Collagen type I alpha 1 chain, EVs: Extracellular vesicles, IFM: Interfascicular extracellular matrix, SCX: Scleraxis, TDSCs: Tendon-derived stem cells.

## Abbreviations

ADSC	Adipose-tissue-derived stem cells
ACL	Anterior cruciate ligament
3D	Three-dimensional
ECM	Extracellular matrix
EVs	Extracellular vesicles
MSCs	Mesenchymal stem cells
TE	Tissue engineering
3R	Replace, reduce, refine
